# Spontaneous Fluctuations in Oscillatory Brain State Cause Differences in Transcranial Magnetic Stimulation Effects Within and Between Individuals

**DOI:** 10.3389/fnhum.2021.802244

**Published:** 2021-12-02

**Authors:** Shanice E. W. Janssens, Alexander T. Sack

**Affiliations:** ^1^Section Brain Stimulation and Cognition, Department of Cognitive Neuroscience, Faculty of Psychology and Neuroscience, Maastricht University, Maastricht, Netherlands; ^2^Maastricht Brain Imaging Centre (MBIC), Maastricht, Netherlands; ^3^Department of Psychiatry and Neuropsychology, School for Mental Health and Neuroscience (MHeNs), Brain + Nerve Centre, Maastricht University Medical Centre+ (MUMC+), Maastricht, Netherlands; ^4^Centre for Integrative Neuroscience (CIN), Maastricht University, Maastricht, Netherlands

**Keywords:** transcranial magnetic stimulation (TMS), inter-and intra-subject variability, neuronal oscillations, multimodal TMS, closed-loop TMS

## Abstract

Transcranial magnetic stimulation (TMS) can cause measurable effects on neural activity and behavioral performance in healthy volunteers. In addition, TMS is increasingly used in clinical practice for treating various neuropsychiatric disorders. Unfortunately, TMS-induced effects show large intra- and inter-subject variability, hindering its reliability, and efficacy. One possible source of this variability may be the spontaneous fluctuations of neuronal oscillations. We present recent studies using multimodal TMS including TMS-EMG (electromyography), TMS-tACS (transcranial alternating current stimulation), and concurrent TMS-EEG-fMRI (electroencephalography, functional magnetic resonance imaging), to evaluate how individual oscillatory brain state affects TMS signal propagation within targeted networks. We demonstrate how the spontaneous oscillatory state at the time of TMS influences both immediate and longer-lasting TMS effects. These findings indicate that at least part of the variability in TMS efficacy may be attributable to the current practice of ignoring (spontaneous) oscillatory fluctuations during TMS. Ignoring this state-dependent spread of activity may cause great individual variability which so far is poorly understood and has proven impossible to control. We therefore also compare two technical solutions to directly account for oscillatory state during TMS, namely, to use (a) tACS to externally control these oscillatory states and then apply TMS at the optimal (controlled) brain state, or (b) oscillatory state-triggered TMS (closed-loop TMS). The described multimodal TMS approaches are paramount for establishing more robust TMS effects, and to allow enhanced control over the individual outcome of TMS interventions aimed at modulating information flow in the brain to achieve desirable changes in cognition, mood, and behavior.

## Introduction

Barker et al. (1985) were the first to show that the human brain could be stimulated non-invasively using rapidly changing magnetic fields. This *transcranial magnetic stimulation* (TMS) method was virtually painless, required minimal preparation, and offered a flexible stimulation coil which could be rapidly and easily moved between scalp locations (brain areas). When the TMS coil was placed on the scalp above the motor cortex, movements could be induced in contralateral body parts, and the muscles’ responses could be measured using electromyography (EMG) (Rothwell et al., 1999; Hallett, 2000, 2007). These so-called “motor-evoked potentials” (MEPs) are caused by the excitation of corticospinal neurons (Berardelli et al., 1990; Burke et al., 1993; Di Lazzaro et al., 1998), and MEPs are still used in contemporary research as a measure of motor cortex excitability (Boroojerdi et al., 2002; Rossini et al., 2015).

Given its ability to directly influence brain processing (Romero et al., 2019), TMS can serve several purposes. It can be used to investigate whether and when a brain area is causally relevant for a cognitive function (Sack, 2006; Sack et al., 2006; de Graaf et al., 2009, 2015; Schuhmann et al., 2009; Jacobs et al., 2012b), and to map the brain’s functional connectivity profile (Pascual-Leone et al., 2000; Sack and Linden, 2003; Sack et al., 2007; Bäumer et al., 2009; de Graaf et al., 2009, 2012; Reithler et al., 2011; Arai et al., 2012). Since its development, TMS has therefore been widely used in cognitive neuroscience research (Walsh and Cowey, 2000; O’Shea and Walsh, 2007), not only to map the motor cortex (Gunduz et al., 2020), but also to study domains such as visual perception (Amassian et al., 1989; Kammer, 2007; Jacobs et al., 2012a, 2014; de Graaf et al., 2014; de Graaf and Sack, 2014; Janssens et al., 2020b), attention (Ashbridge et al., 1997; Sack et al., 2002, 2007; Rushworth and Taylor, 2006; Ronconi et al., 2014; Duecker and Sack, 2015), imagery (Sack et al., 2002, 2005; Cattaneo et al., 2012), language (Pascual-Leone et al., 1991; Schuhmann et al., 2012; Acheson and Hagoort, 2013; Tarapore et al., 2013), learning (de Weerd et al., 2012; Platz et al., 2012a,b), and memory (Osaka et al., 2007; van de Ven et al., 2012; van de Ven and Sack, 2013; Bonnì et al., 2015; Rademaker et al., 2017; Ferrari et al., 2018). In addition, TMS is increasingly used in clinical practice for treating various neuropsychiatric disorders (Lefaucheur et al., 2014, 2020; de Graaf et al., 2021a,b). TMS is used during stroke rehabilitation (Hummel and Cohen, 2006; Di Pino et al., 2014; Wessel et al., 2015), and as treatment for depression (Loo and Mitchell, 2005; Perera et al., 2016; Donse et al., 2018; Baeken et al., 2019; Sonmez et al., 2019) and schizophrenia (Cole et al., 2015).

## Immediate and Aftereffects of Transcranial Magnetic Stimulation Show High Inter- and Intra-Subject Variability

Given the widespread use of TMS in research and clinical settings, one might assume that TMS generally leads to positive and consistent findings. Yet, the effects of TMS are not always robust and reliable. Inconsistent TMS effects between experiments/clinical trials could partially be due to methodological factors, such as differences in the coil placement method (Beam et al., 2009; Rusjan et al., 2010; Gomez et al., 2021). But even if methodological factors are kept constant, TMS effects can show substantial variability. There are two types of variability in the effects of TMS: different individuals may respond differently to TMS (*inter-subject variability*), and the effect of TMS may differ within the same individual over time (*intra-subject variability*). We should furthermore distinguish between two types of TMS effects: the *immediate* effects of single-pulse TMS, and the *aftereffects* of repetitive TMS (“rTMS”). Below, we present evidence that suggests that both the immediate and aftereffects of TMS show substantial inter- and intra-individual variability.

The *immediate* effects of single-pulse TMS to the primary motor cortex are often measured with MEPs, which provide a measure of the momentary TMS reactivity (Rossini et al., 2015). Within the same individual, TMS-MEP amplitudes vary over trials (Kiers et al., 1993; Burke et al., 1995; Wassermann, 2002; Rösler et al., 2008; Goetz et al., 2014; Goldsworthy et al., 2016a). Interestingly, optimization of TMS target localization does not necessarily improve the variability and reproducibility of TMS-induced MEPs (Jung et al., 2010). This finding already suggests that factors beyond the TMS parameters may contribute to immediate TMS reactivity.

Such variability in immediate TMS effects is not limited to the motor network. When stimulating early visual cortex, some individuals can perceive “phosphenes” (an illusory percept). The “phosphene threshold” (the minimal TMS intensity required to perceive a phosphene in half of the cases) is often used as a measure of visual cortex excitability (Boroojerdi et al., 2002; Bestmann et al., 2007; de Graaf et al., 2017). The probability of inducing phosphenes within the same participant can vary over time (Gerwig et al., 2003; Romei et al., 2008a, b; Dugué et al., 2011).

Variability in TMS *aftereffects* can be illustrated by evaluating individual responses to rTMS protocols that were designed to modulate synaptic plasticity beyond the duration of stimulation (Pascual-Leone et al., 1998; Ridding and Ziemann, 2010). Low (<1 Hz) and high (>1 Hz) frequency rTMS were originally reported to decrease and increase the excitability of the human motor cortex, respectively (Wassermann et al., 1998). This may indeed be the case on average, but when inspecting individual responses, not all participants showed these effects (Maeda et al., 2000). Similarly, intermittent and continuous theta burst stimulation (iTBS and cTBS, two forms of patterned rTMS) were reported to enhance and suppress motor cortex excitability for ∼30 min after stimulation, respectively (Huang et al., 2005). These findings have not always been replicated in another subject sample (Goldsworthy et al., 2012; Hordacre et al., 2017), and even if they are present at the group level, not all individuals show these effects (Cheeran et al., 2008; Nettekoven et al., 2015; Schilberg et al., 2017). In fact, one study reported that only 1 in 4 participants showed the expected pattern of results (Hamada et al., 2013). Another TMS procedure aimed at modulating neuroplasticity is called “paired associative stimulation” (PAS). Originally, PAS involved peripheral nerve stimulation that was paired with single-pulse TMS to primary motor cortex in order to enhance corticomotor excitability (Stefan et al., 2000), but PAS has also been employed to facilitate communication between the motor cortex and interconnected cortical areas (Veniero et al., 2013). As for the other plasticity-inducing TMS protocols, there is high inter-subject variability in the effects of PAS (Sale et al., 2007; Florian et al., 2008; López-Alonso et al., 2014), with a recent study reporting that only 61% of participants responded to PAS (Minkova et al., 2019).

Besides inter-subject variability, TMS aftereffects also show significant intra-subject variability. Some reports indicated that the aftereffects of iTBS and cTBS were relatively stable within the same individuals (Hinder et al., 2014; Vernet et al., 2014), but a recent study showed the opposite (Schilberg et al., 2017). Schilberg et al. (2017) further investigated the within-subject reliability of iTBS effects over the course of 60 min, and across two experimental sessions that were scheduled ∼8 days apart. They found that the effect of iTBS on corticospinal excitability (as measured with MEP amplitude) differed between sessions. The average increase in MEP amplitude was approximately 23% in the first session, but only approximately 6% during the second visit.

From these examples, it becomes clear that TMS effects show considerable inter- and intra-subject variability, for both the immediate effects of single-pulse TMS (MEP amplitudes, phosphene induction) and the longer-lasting plasticity effects as induced by rTMS, TBS, or PAS. The limited consistency of TMS effects can have negative consequences in research and clinical settings, because TMS effects are not always predictable or optimized. If TMS effects are not sufficiently reliable, they thus have limited use as a biomarker for individual changes in neuroplasticity and concomitant desirable changes in cognition and behavior (Schambra et al., 2015). It is therefore important to identify the factors that contribute to the variability of TMS effects (Corp et al., 2020, 2021), such that the consistency and efficacy of TMS can be improved. We here discuss one possible source of this variance, namely, spontaneous fluctuations in neuronal oscillations (Buzsáki and Draguhn, 2004; Pasley et al., 2009; Iscan et al., 2016; Bergmann, 2018). Below, we explain how spontaneous fluctuations in oscillatory brain state contribute to variability both in the immediate effects of TMS and in TMS-induced plasticity effects.

## Spontaneous Fluctuations in Neuronal Oscillations Contribute to Variations in Immediate Transcranial Magnetic Stimulation Effects

To investigate the link between TMS effect variability and ongoing neuronal oscillations, TMS can be combined with magneto- or electroencephalography (M/EEG). Specific characteristics of neuronal oscillations (i.e., their frequency, power, or phase; Palva and Palva, 2007) might be correlated with the immediate responsivity to single-pulse TMS. Indeed, the probability of inducing phosphenes when applying TMS to early visual cortex was negatively correlated with EEG alpha power prior to TMS (Romei et al., 2008a,b). The probability of perceiving TMS-induced phosphenes was also associated with the phase of ongoing EEG alpha oscillations (Dugué et al., 2011). Results have been less clear for the motor system. Some studies reported a negative association between pre-TMS EEG alpha power and TMS-induced MEP amplitude (Sauseng et al., 2009; Zarkowski et al., 2016). Others reported a negative association between TMS-MEP amplitude and oscillatory beta power (Lepage et al., 2008; Mäki and Ilmoniemi, 2010; Schulz et al., 2014), or no relation with oscillatory power in any frequency band (Mitchell et al., 2007; Berger et al., 2014). Spontaneous fluctuations in the phase of ongoing beta (Keil et al., 2014) and alpha (Schaworonkow et al., 2018, 2019; Bergmann et al., 2019) oscillations may also play a role in TMS-MEP variability. Note that inconsistencies across studies may in part be explained by methodological differences, such as differences in TMS intensity (Pellegrini et al., 2018).

Schilberg et al. (2021) recently assessed the relation between the power and phase of ongoing EEG alpha and beta oscillations with motor cortex TMS reactivity. They found that TMS-MEP amplitude correlated positively with pre-TMS oscillatory power in the alpha and beta bands. The authors also reported a significant effect of alpha phase on TMS-MEP amplitude, but there was no consistent alpha phase that led to high TMS-MEP amplitudes across participants. The latter is in contrast with previous reports showing that higher TMS-induced MEP amplitudes are mostly induced during alpha troughs instead of peaks (Schaworonkow et al., 2018, 2019; Zrenner et al., 2018). Interestingly, a standard FFT analysis did not reveal a significant correlation between pre-TMS beta phase and TMS-MEP amplitude, while a Hilbert transform did show an effect (Schilberg et al., 2021). This discrepancy between analyses may be partially explained by the variability in individual beta frequency (IBF), which is larger than the variability in individual alpha frequency (IAF) (Haegens et al., 2014). The Hilbert transform is less affected by frequency variations compared to the FFT approach, since the former can be used for non-stationary time series (Schilberg et al., 2021). Another contributing factor might be that participants were not involved in any active motor task. Ongoing beta power was therefore naturally low, making it more difficult to reliably estimate beta phase. When TMS is applied at high beta power, the relation between beta phase and TMS-MEP amplitude indeed becomes evident (Torrecillos et al., 2020). In any case, most of the evidence presented above is of correlational nature, because oscillations were measured rather than experimentally manipulated.

## Direct Evidence for a Causal Link Between (Controlled) Oscillatory State and Variations in Immediate Transcranial Magnetic Stimulation Effects

Transcranial alternating current stimulation (tACS) can be used to establish the *causal* relevance of neuronal oscillations (Herrmann et al., 2016). TACS is a form of non-invasive brain stimulation (NIBS) that involves electrical stimulation with a sinusoidal waveform (Antal and Paulus, 2013). It can be used to enhance the power of oscillations of a certain frequency within the stimulated brain area (Herrmann et al., 2013; Vossen et al., 2015; Vieira et al., 2020), potentially through mechanisms of entrainment (Thut et al., 2011; Huang et al., 2021) or spike-timing dependent plasticity (Herrmann et al., 2013; Vossen et al., 2015). The causal relevance of oscillatory phase can then be established by presenting stimuli at certain phases of the tACS waveform (de Graaf et al., 2020). It was previously shown that it is possible to apply TMS at certain tACS phases with high temporal precision (ten Oever et al., 2016), and that it is feasible to use simultaneous tACS-TMS to investigate the causal relation between oscillatory tACS phase and TMS-MEP amplitudes (Raco et al., 2016). The same logic was applied by Schilberg et al. (2018), who administered TMS pulses at eight equidistant phases of a tACS waveform, using IBF-, IAF-, or sham tACS to primary motor cortex. The authors found that tACS modulated TMS-MEP amplitude only for the IBF-tACS condition, and this effect seemed to be specific to individuals with lower IBF frequencies. These findings suggest that beta-tACS phase at the time of TMS influences the immediate effects of TMS (*intra-subject variability*), and that this effect interacts with the individual dominant beta frequency (*between-subject variability*) (Haegens et al., 2014).

## Spontaneous Fluctuations in Neuronal Oscillations Contribute to the Propagation of Transcranial Magnetic Stimulation Pulses Through Functionally Connected Networks

Simultaneously combining TMS with M/EEG or tACS is an excellent approach to investigate the link between ongoing neuronal oscillations and the variability of TMS effects. However, this approach does not allow an accurate (high-resolution) visualization of the immediate effects of TMS at the level of the brain. Functional magnetic resonance imaging (fMRI) can be used to visualize TMS signal propagation, given its potential to measure whole-brain activation with good spatial resolution (Walsh and Cowey, 2000; Sack and Linden, 2003; Sack, 2006; Bestmann et al., 2008; Reithler et al., 2011). Simultaneous TMS-fMRI studies have shown that the effects of TMS pulses can extend beyond the targeted brain area, since signals can spread toward interconnected brain areas (Ruff et al., 2006; Sack et al., 2007; Blankenburg et al., 2010). Though the local effects of TMS pulses do not reach deeper than the superficial cortex, remote effects can even be observed in subcortical areas (Bergmann et al., 2021). Nonetheless, to achieve a full understanding of how TMS pulses propagate through functionally connected networks, it is important to investigate whether and how TMS-evoked fMRI responses vary as a function of ongoing neuronal oscillations on a trial-by-trial level. This was made possible with a unique setup, which simultaneously combines TMS, EEG, and fMRI.

This technically challenging experimental triad approach was introduced by our lab in 2013 (Peters et al., 2013). We demonstrated that concurrent TMS-EEG-fMRI is feasible and safe in both phantom and human measurements, and we showed that the EEG and fMRI data were of sufficient quality. Yet, the full potential of this approach only became apparent in a recent publication from our lab, in which we mapped whole-brain TMS signal propagation as a function of the pre-TMS oscillatory state as indexed by simultaneous EEG (Peters et al., 2020). In four healthy individuals, we applied triple-pulse (15-Hz) TMS to the right dorsal premotor area (PMd), while continuously measuring EEG. Triple-pulse TMS was used to probe the motor network with a sufficiently strong stimulus, rather than to modulate neuroplasticity as with typical rTMS protocols (the findings described here thus relate to *immediate* TMS effects).

TMS to PMd evoked both local and remote fMRI activation in a cortico-subcortical motor network, resembling the activations as seen for voluntary movements. It again became evident that different individuals may respond differently to TMS (*inter-subject variability*): two individuals showed less/more confined activations in response to TMS compared to the other two individuals. These individuals also showed less engagement of the motor network irrespective of TMS (“low activators,” the others were called “high activators”). It should be noted that the difference in TMS-evoked responses may in part be due to differences in TMS intensity between the “low activators” and “high activators.” In any case, to evaluate immediate TMS-evoked responses within the cortico-subcortical motor network as a function of oscillatory state, it was crucial that participants showed reliable engagement of the motor network. The EEG-informed analyses were therefore performed only for the two “high activators.”

The main question of interest was whether TMS signal propagation within a cortico-subcortical motor network varies with pre-TMS parietal alpha power. Pre-TMS alpha power was negatively correlated with TMS-evoked fMRI responses in both local and remote (including subcortical) areas of the motor network. This negative association is in line with the supposed inhibitory role of alpha oscillations (Klimesch et al., 2007). From these findings, we can conclude that, within the same individual, TMS pulses may propagate differently throughout the motor network depending on pre-TMS oscillatory state (*intra-subject variability*). Our group has recently also established the feasibility of using simultaneous TMS-EEG-fMRI for non-motor areas (Janssens et al., 2020a). This comes with additional technical challenges, including the determination of the TMS site and intensity, because most non-motor areas are so-called “silent” areas that do not show any overt response to TMS.

## Direct Evidence for a Causal Link Between (Controlled) Oscillatory State and Variations in Transcranial Magnetic Stimulation Aftereffects

Thus far, we focused on within- and between-subject variability in the *immediate* effects of TMS, and how such variability can be linked to ongoing neuronal oscillations. There is reason to believe that changes in oscillatory state also contribute to variations in TMS-induced neuroplasticity (TMS *aftereffects*). Goldsworthy et al. (2016b) applied cTBS to the primary motor cortex, while phase-aligning the TMS pulses to either the peak or the trough of concurrent alpha-tACS. They investigated whether the response to cTBS, as measured with TMS-induced MEP amplitudes, depended on the alpha-tACS phase. The excitability of the motor cortex was suppressed (TMS-MEP amplitudes were reduced) when cTBS was aligned with alpha-tACS troughs. Crucially, cTBS did not modulate motor cortex plasticity when cTBS was aligned with alpha-tACS peaks. Furthermore, the effect of tACS-trough-aligned cTBS was greater for individuals with higher IAFs (Goldsworthy et al., 2016b). Thus, TMS-induced neuroplasticity may vary both as a function of the controlled momentary oscillatory state and the intrinsic dominant oscillatory frequency.

Besides oscillatory phase, the power of ongoing neuronal oscillations might be relevant for TMS-induced neuroplasticity as well. Guerra et al. (2018) showed that concurrent gamma tACS enhanced and prolonged iTBS-induced increases in TMS-MEP amplitude, in contrast to beta-tACS and sham-tACS (Guerra et al., 2018). This positive effect of simultaneous gamma tACS on iTBS efficacy was later replicated, but it seems that simultaneous gamma tACS *reduced* the efficacy of cTBS (Guerra et al., 2020a). These findings are especially relevant in a clinical context, where the goal is to employ rTMS to modulate neuroplasticity for longer periods of time. It would be beneficial to optimize plasticity-inducing TMS protocols based on oscillatory brain state, such that treatment efficacy can be improved.

## Accounting for Spontaneous Fluctuations in Neuronal Oscillations During Transcranial Magnetic Stimulation

Thus far, we have outlined that immediate and prolonged TMS effects vary considerably within- and between-individuals. We also showed that spontaneous fluctuations in neuronal oscillations can explain at least part of the variability in TMS effects, as can more stable oscillatory characteristics (individual peak frequencies). The question then becomes: how can we incorporate such oscillatory information into our TMS protocols?

The first step is to form a clear hypothesis regarding the to-be-targeted oscillatory frequency, since different frequency bands are associated with different functions (Başar et al., 1999; Ward, 2003; Clayton et al., 2018). Even within the same (e.g., alpha) frequency band, there might be different functionally relevant oscillation generators in the brain, which are not easily disentangled in the M/EEG signal (Bollimunta et al., 2011; Haegens et al., 2015; Sokoliuk et al., 2019). More advanced techniques might be needed to extract the relevant oscillatory frequency from the M/EEG signal (Schaworonkow et al., 2018). Once the relevant oscillatory frequency has been determined, there are two potential technical solutions that can directly account for oscillatory brain state during TMS: simultaneous tACS-TMS, and M/EEG-based “closed-loop” TMS (Huang et al., 2017).

As discussed previously, TMS can be applied at the (controlled) optimal tACS phase (Raco et al., 2016; ten Oever et al., 2016; Fehér et al., 2017). Crucially, individuals differ in terms of their oscillatory brain rhythms. For instance, peak alpha frequencies (IAFs) can range between 7 and 14 Hz across individuals (Haegens et al., 2014). To ensure optimal tACS efficacy, it is therefore important to individually calibrate the tACS frequency, for instance based on a resting state M/EEG measurement (Janssens et al., 2021) or through functional identification (Gundlach et al., 2017; Schilberg et al., 2018). Besides personalizing the tACS frequency, it might also be necessary to individually determine the optimal tACS phase to deliver TMS, given the recent finding that no consistent alpha phase was correlated to high TMS-MEP amplitudes (i.e., high TMS responsivity) across participants (Schilberg et al., 2021). Simultaneous tACS-TMS has already been used to link tACS beta phase to motor cortex TMS reactivity (Guerra et al., 2016; Schilberg et al., 2018). It has furthermore been shown that single TMS pulses applied to dorsolateral prefrontal cortex propagate differently through a cortical network depending on the phase of concurrent theta-tACS (Fehér et al., 2017). Thus, by applying single-pulse TMS at the optimal (controlled) tACS phase, TMS signal propagation may be modulated. Besides its relevance for immediate TMS effects, tACS can also be used to enhance and prolong TMS aftereffects, as described above (Goldsworthy et al., 2016b; Guerra et al., 2018, 2020a).

Simultaneous tACS-TMS is useful, but not perfect. Individual peak frequencies show good within-subject test-retest reliability (Grandy et al., 2013; Haegens et al., 2014; Janssens et al., 2021), but peak frequencies can still fluctuate, and the extent to which this happens differs across individuals. For example, IAF decreased over the course of 1 h during visual task performance, with some participants showing reductions of up to 2 Hz (Benwell et al., 2019). If tACS were to be applied at the originally determined peak frequency, tACS efficacy may be compromised, since the matching between the endogenous dominant frequency and the driving (tACS) frequency would not always be optimal (Romei et al., 2016). The best approach might thus be to continuously track the instantaneous dominant frequency, and to adjust the tACS frequency accordingly. However, it is difficult to recover EEG signals during tACS due to the sizeable tACS artifacts (Kasten and Herrmann, 2019). Another complication of the simultaneous tACS-TMS approach is that if the effect of tACS on oscillatory activity is not verified through means of concurrent M/EEG measurements, we cannot be certain that the applied tACS phase corresponds to the phase of ongoing neuronal oscillations. Finally, it could be the case that there is an “optimal” amount of oscillatory power, in the sense that if tACS enhances oscillatory power above a certain threshold, it might *reduce* the reactivity of a brain area to TMS.

In contrast to simultaneous tACS-TMS, the second technical solution to account for oscillatory brain state during TMS *does* measure ongoing neuronal oscillations. In this so-called “closed-loop” TMS approach, the M/EEG signal is continuously measured, and the timing of TMS pulses is adjusted to the optimal power and/or phase of the ongoing oscillations (Bergmann et al., 2016; Zrenner et al., 2016; Thut et al., 2017; Guerra et al., 2020b). This method can only be successful if the instantaneous phase can be reliably estimated (that is, if the power of the ongoing oscillations is sufficiently high). This has two important implications if the aim is to target specific oscillatory phases. Firstly, it might be necessary to control participants’ cognitive state (i.e., task engagement vs. rest) to ensure high oscillatory power. Secondly, the closed-loop TMS approach might fail in individuals that show naturally/pathologically low oscillatory power.

Irrespective of these technical challenges, EEG-based closed-loop TMS has already been applied successfully. It was shown that MEP amplitudes were higher during the rising phase of ongoing slow (<1 Hz) oscillations compared to the falling phase, when TMS was applied to primary motor cortex (Bergmann et al., 2012). Interestingly, these findings were consistent across two cognitive states (wakefulness and sleep). In another study, rTMS applied to primary motor cortex at the troughs of the ongoing alpha rhythm enhanced MEP amplitudes, while rTMS applied at alpha peaks did not (Zrenner et al., 2018). These findings clearly show that temporally targeting TMS pulses to the optimal oscillatory state improves its efficacy both in terms of signal propagation (immediate effects) and the induction of neuroplasticity (aftereffects).

## Conclusion

TMS is widely used in both research and clinical settings. Still, its immediate and prolonged effects are not robust and reliable, as is evident from both intra- and inter-subject variability. One potential source of this variability may be the spontaneous fluctuations of neuronal oscillations. We showed this for both immediate TMS effects (TMS-MEP amplitudes, TMS phosphene induction, TMS-fMRI signal propagation), and for TMS aftereffects (of rTMS, TBS, or PAS). The oscillatory brain state can be accounted for during TMS by using either simultaneous tACS-TMS or closed-loop M/EEG-TMS. This may reduce both inter- and intra-individual variability in TMS effects. The described multimodal TMS approaches allow enhanced control over the individual outcome of TMS protocols aimed at modulating information flow and/or neuronal plasticity in the healthy and diseased brain. They therefore pave the way to stronger and more consistent TMS-induced improvements in cognition, mood, and behavior.

## Author Contributions

SJ: conceptualization, writing—original draft, writing—review and editing, and funding acquisition. AS: conceptualization, writing—review and editing, funding acquisition, and supervision. Both authors contributed to the article and approved the submitted version.

## Conflict of Interest

The authors declare that the research was conducted in the absence of any commercial or financial relationships that could be construed as a potential conflict of interest.

## Publisher’s Note

All claims expressed in this article are solely those of the authors and do not necessarily represent those of their affiliated organizations, or those of the publisher, the editors and the reviewers. Any product that may be evaluated in this article, or claim that may be made by its manufacturer, is not guaranteed or endorsed by the publisher.
